# Evaluating an LLM-assisted workflow for clinical documentation: A pilot randomized controlled trial on time and quality

**DOI:** 10.1371/journal.pdig.0001799

**Published:** 2026-10-26

**Authors:** Tomohiro Takayama, Keina Sado, Kenji Suda, Hiroshi Tamura, Naoko Ueda-Arakawa, Kenji Ishihara, Yuta Ueda, Kazuya Okamoto, Luciano Henrique de Oliveira Santos, Tetsuro Oshika, Tomohiro Kuroda, Akitaka Tsujikawa, Masahiro Miyake

**Affiliations:** 1 Department of Ophthalmology and Visual Sciences, Kyoto University Graduate School of Medicine, Kyoto, Japan; 2 Kyoto University Hospital, Kyoto, Japan; 3 Fitting Cloud Inc., Kyoto, Japan; 4 Center for Innovative Research and Education in Data Science, Institute for Liberal Arts and Sciences, Kyoto University, Kyoto, Japan; 5 Japanese Society of Artificial Intelligence in Ophthalmology, Chuoku, Tokyo, Japan; 6 Department of Ophthalmology, Faculty of Medicine, University of Tsukuba, Ibaraki, Japan; 7 Division of Medical Information Technology and Administration Planning, Kyoto University Hospital, Kyoto University, Kyoto, Japan; UMC Utrecht: Universitair Medisch Centrum Utrecht, NETHERLANDS, KINGDOM OF THE

## Abstract

Large language models (LLMs) have been investigated for clinical documentation, with concerns about hallucinations and factual errors. Clinician review and revision of LLM-generated drafts are considered essential; however, the impact of such a workflow on both documentation time and quality remains unknown. This single-center, parallel-group, prospective, randomized, open-label, blinded-endpoint (PROBE) pilot trial evaluated whether a clinician-in-the-loop workflow improves clinical documentation time and quality compared with clinician-only drafting. From February 18 to March 14, 2025, at the Department of Ophthalmology, Kyoto University Hospital, 21 physicians were randomized, and 17 completed the intervention. Participants were randomized to either the Clinician-in-the-loop group or the Clinician-only group. Participants created discharge summaries and referrals for six simulated patient records. The Clinician-in-the-loop group reviewed and edited LLM-generated drafts, whereas the Clinician-only group drafted documents from scratch using matched templates. Unedited LLM drafts formed the LLM-only group. Seventeen physicians submitted 48 discharge summaries and 48 discharge referrals in the Clinician-in-the-loop group, 54 of each document type in the Clinician-only group, and 48 of each in the LLM-only group. Clinician-in-the-loop showed a shorter creation time for summaries (β = −59.4 seconds; 95% CI, −150.6 to 31.7; P = .18) and a longer creation time for referrals (β = 52.9 seconds; 95% CI, −7.0 to 112.9; P = .08) than Clinician-only, although neither difference was statistically significant. In most expert-rated quality domains, the clinician-in-the-loop workflow outperformed clinician-only drafting. LLM-only drafts were fastest but had the lowest quality. In conclusion, a clinician-in-the-loop approach generally improved document quality and, although the primary analysis showed no statistically significant difference in documentation time, suggested potential time savings for certain document types. These findings highlight the importance of active clinician supervision and support further evaluation of clinician-in-the-loop workflows in real-world clinical settings.

Trial Registration

ClinicalTrials.gov Identifier NCT07187050 (registered on September 18, 2025).

## Author summary

Clinical documentation such as discharge summaries and discharge referrals is essential for patient care, yet it places a heavy burden on clinicians. Recently, large language models (LLMs) have been proposed as tools that might ease this burden, but it remains uncertain whether documents created with the help of an LLM can save time while still maintaining acceptable quality. In this study, we conducted a randomized controlled trial in which clinicians created clinical documents either by reviewing and editing drafts generated by an LLM or by writing them on their own. We also analyzed unedited LLM drafts to understand how the model performs without clinician review. We found that the clinician-in-the-loop approach generally improved document quality and may save time for some document types, although the primary analysis found no statistically significant difference in documentation time. In contrast, LLM-only drafts were produced most quickly but did not meet quality standards. These findings suggest that LLMs may support clinical documentation when paired with active clinician oversight. Such collaborative workflows warrant further evaluation in real-world clinical settings.

## Introduction

Clinicians are significantly involved in clerical tasks, especially electronic health record (EHR) documentation. A large U.S. time-motion study revealed that approximately 49% of physicians’ total working time is spent on EHR use and other desk work, with documentation alone accounting for roughly one-third of each patient encounter [1]. Similarly, a survey of physicians at 80 Japanese hospitals revealed that EHR-related documentation consumed 13% of weekly working hours and represented one of the strongest predictors of overtime [2]. This documentation workload not only extends working hours but also contributes to diminished job satisfaction and greater risk of burnout among clinicians [3–6].

In this context, large language models (LLMs) have been proposed to alleviate this burden [7] and have received increasing attention because they can help simplify administrative tasks in healthcare [8,9]. Recent investigations have applied models such as GPT‑4 and Claude to the automated drafting of discharge summaries [10–18], discharge referrals [16,19], radiology reports [20,21], or other types of medical documents [20,22–26]. However, comparative evaluations consistently report significant limitations. These include hallucinated findings [11,13,23], factual omissions [23], factual errors [11–13,23,24], and occasionally harmful recommendations [13,21,24], which lead reviewers to conclude that LLM-generated documents are not sufficiently reliable for clinical practice [13,21,24].

As hallucinations and other factual inaccuracies are clinically unacceptable, LLM-generated drafts must be verified and rectified, if required, by clinicians [15,22,27]. Accordingly, evaluation of the clinical utility of LLMs for document creation should consider the entire documentation process—a clinician-in-the-loop workflow that includes clinician review and editing after generation [18,27]. However, most existing studies have assessed model output in isolation; to our knowledge, none has jointly evaluated time and quality within the workflow.

In the current study, to evaluate this clinician-in-the-loop workflow, we conducted a pilot randomized controlled trial using a novel LLM assistant, CocktailAI. This trial assessed documentation time and quality by comparing three approaches: documents drafted by our LLM assistant and then modified by clinicians, documents created entirely by clinicians, and documents generated by the LLM assistant with no human review.

## Materials and methods

### Ethics statement

This study was conducted in the context of an existing collaborative research agreement between Kyoto University Hospital and Fitting Cloud Inc. The Institutional Review Board of FINDEX Inc., the parent company of Fitting Cloud Inc., ruled that approval was not required for this study. Written informed consent was obtained from all participating physicians. The study adhered to the tenets of the Declaration of Helsinki.

### Study design and setting

This single-center, parallel-group, prospective, randomized, open-label, blinded-endpoint (PROBE) trial was conducted at Kyoto University Hospital to evaluate the impact of the clinician-in-the-loop workflow. This study was reported in accordance with the Consolidated Standards of Reporting Trials (CONSORT) reporting guideline for randomized clinical trials [28], and the completed CONSORT checklist is provided as supporting information. The trial was retrospectively registered at ClinicalTrials.gov (Identifier: NCT07187050) on September 18, 2025. At the time of study initiation, trial registration was not considered mandatory because this was a pilot digital-health randomized trial involving healthcare professionals rather than patients and evaluating provider-side workflow outcomes. Nevertheless, to enhance transparency, the study was subsequently registered. The study protocol, outcomes, and statistical analysis plan were finalized before participant enrollment and are provided in S1 Protocol.

Participants were randomized to either an LLM-assisted documentation group (Clinician-in-the-loop group) or a manual, from-scratch documentation group (Clinician-only group). Randomization was performed using permuted block randomization with a block size of two and a 1:1 allocation ratio, stratified by participants’ position. The randomization list was generated in advance using an online tool (https://www.sealedenvelope.com/simple-randomiser/v1/lists), and participants were assigned sequentially according to this list. The study was conducted from February 18 to March 14, 2025. Participants were blinded to their group assignments prior to the initiation of the intervention; however, both participants and investigators were unblinded during the intervention phase. Outcome assessors evaluating the quality of the clinical documents were blinded to group assignments.

### Participants and recruitment

Eligible participants included junior residents, senior residents, and attending ophthalmologists in the Department of Ophthalmology at Kyoto University Hospital. Physicians were eligible if they were actively working in the department during the study period and provided the confirmation that they did not routinely utilize CocktailAI for clinical documentation. The recruitment and allocation process is illustrated in Fig 1.

**Fig 1:**
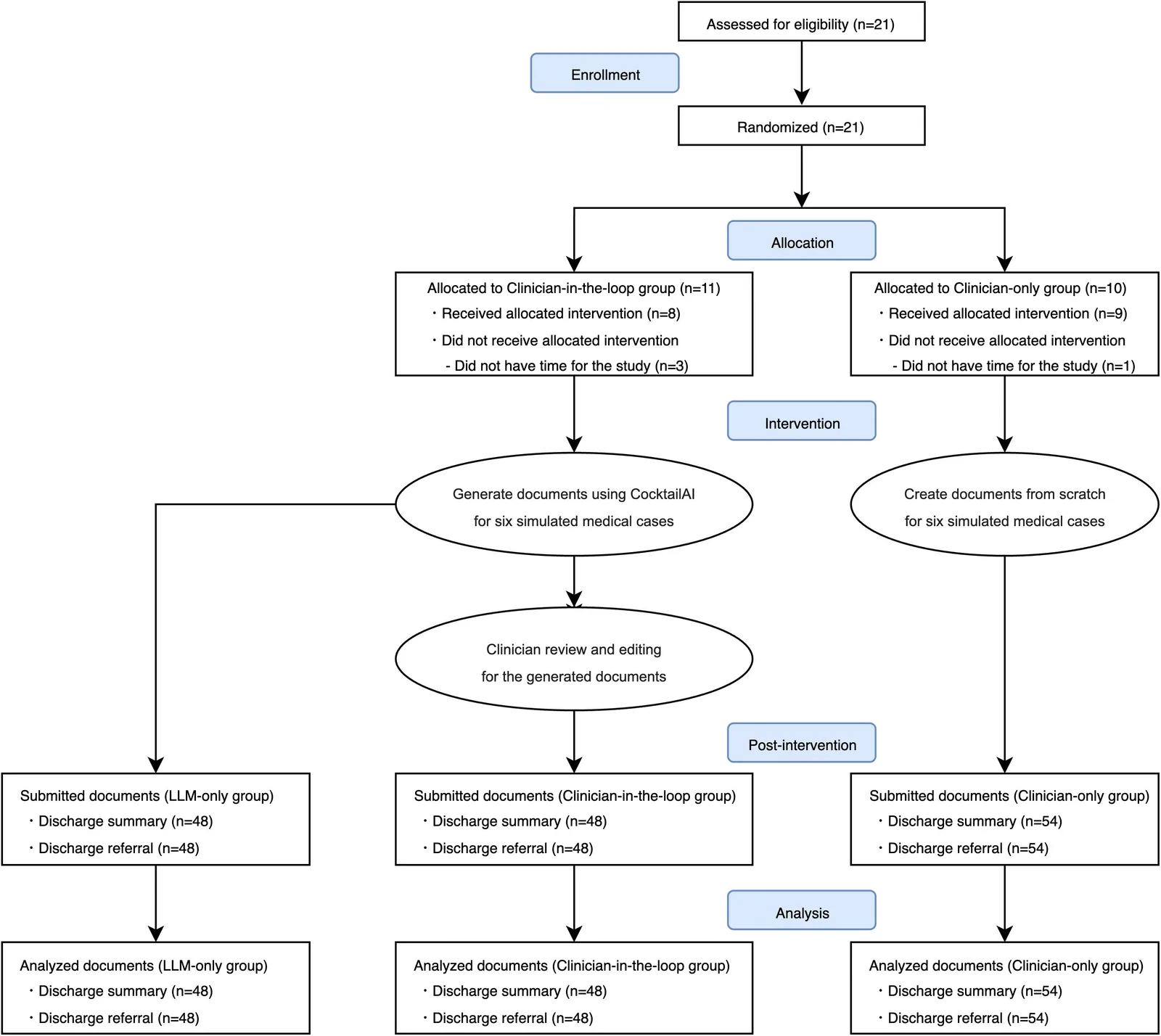
CONSORT flow diagram. A CONSORT-style flow diagram illustrating the enrollment, allocation, post-intervention, and analysis of participants.

The study period was February 18–March 14, 2025. The investigators explained the study overview and eligibility criteria to the participants and obtained written informed consent before any study procedures. Participants then completed a baseline questionnaire capturing demographic and professional characteristics, including age, sex, position, years of clinical experience, average daily time spent using EHRs, average number of days per week using EHRs, and the frequency of generative AI use.

### Simulated medical record

Six simulated patient records were developed in Japanese by Keina S. and Kenji S., representing diverse ophthalmology cases: cataract, ocular trauma, retinal detachment, corneal ulcer, optic neuritis, and glaucoma. Each record comprised comprehensive inpatient demographic data, pre-admission outpatient SOAP notes, and daily inpatient SOAP notes. The simulated records were formally approved by the Japanese Society of Artificial Intelligence in Ophthalmology as suitable for evaluation. English translations of all cases are provided in S1 Appendix.

### Evaluation system

To precisely measure the time required for drafting and subsequent clinician modifications, we developed a purpose-built web application system that reproduces the clinical documentation workflow. In this system, CocktailAI, co-developed by the Department of Ophthalmology and Visual Sciences at Kyoto University Graduate School of Medicine and Fitting Cloud Inc. (Kyoto, Japan), served as the LLM assistant. It assists in the creation of clinical documents by extracting relevant information from EHRs using LLMs and embedding the extracted content into predefined templates (Fig 2). In this study, the system employed the generative AI model Gemini-2.0-flash-lite via an API interface. Because the task was constrained to structured data extraction rather than open-ended generation, the use of a relatively large language model was not considered necessary to achieve reliable performance. Furthermore, at the time of this study, CocktailAI had already been implemented within the electronic health record system. As it was intended for routine clinical use, a smaller model was selected to ensure shorter response times. Output was structured and enforced in JSON format. The model was configured with a temperature of 0 and a top-p value of 0.95. Because CocktailAI is proprietary commercial software, we cannot publicly release the source code related to API integration or backend implementation.

**Fig 2:**
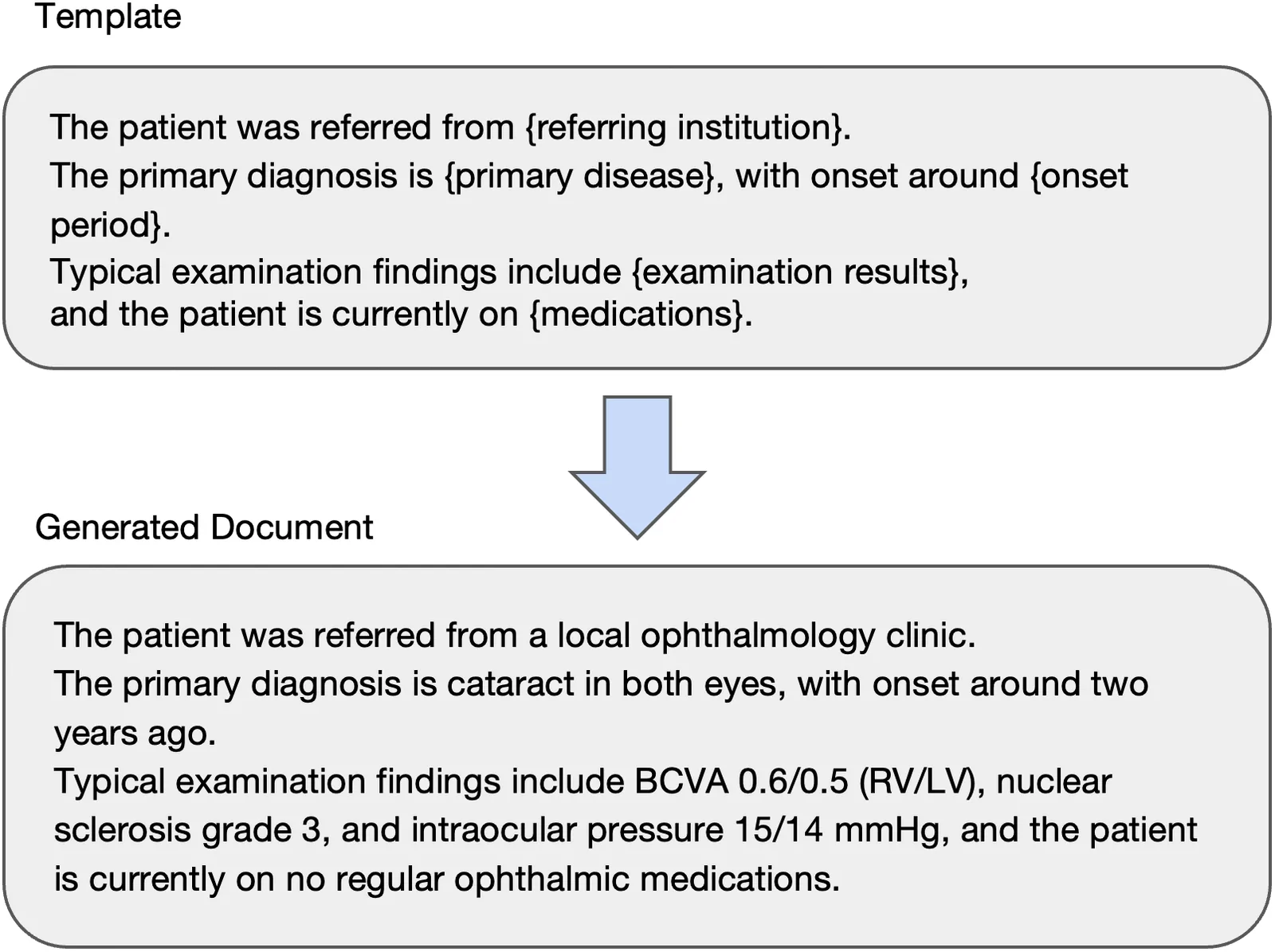
Example of template-based document generation. An LLM assistant was developed collaboratively by the Department of Ophthalmology and Visual Sciences at Kyoto University Graduate School of Medicine, and Fitting Cloud Inc. It extracts relevant information from EHRs and generates draft texts using predefined templates. Segments in {…} act as machine-readable directives, guiding the LLM assistant to retrieve the specified information directly from the EHR.

The templates applied in this study were created by T.T., who was not involved in developing the simulated patient records. They were based on recent discharge summaries and discharge referrals from the Department of Ophthalmology. The complete content of each template is provided in S2 Appendix.

### Study arms

We tasked participants with creating two documents: discharge summary and discharge referral, for each of six simulated patient cases under two groups: the Clinician-in-the-loop group and the Clinician-only group. For each simulated case, participants created a discharge summary first and then a discharge referral; both the task order and the sequence of the six cases were fixed and identical for all participants.

In the Clinician-in-the-loop group, participants first generated a draft with the LLM assistant based on the predefined templates. Thereafter, they reviewed the LLM-generated text and edited it. Copy-and-paste from the patient record was permitted. No regeneration of LLM drafts was allowed.

In the Clinician-only group, participants worked from the same six patient records and completed the same two documents but had no access to the LLM assistant. They applied templates with the same structure as those in the Clinician-in-the-loop group, although all LLM instruction prompts had been removed in advance to avoid the additional time required to delete them for each use. Copy-and-paste from the record was also allowed.

Furthermore, an LLM-only group was included among the study groups. We retained the unedited drafts automatically produced by the LLM assistant before any human review or modification in the Clinician-in-the-loop group. These outputs represented LLM-generated documents without clinician review and were analyzed as a separate group. The above information is presented in Fig 3.

**Fig 3:**
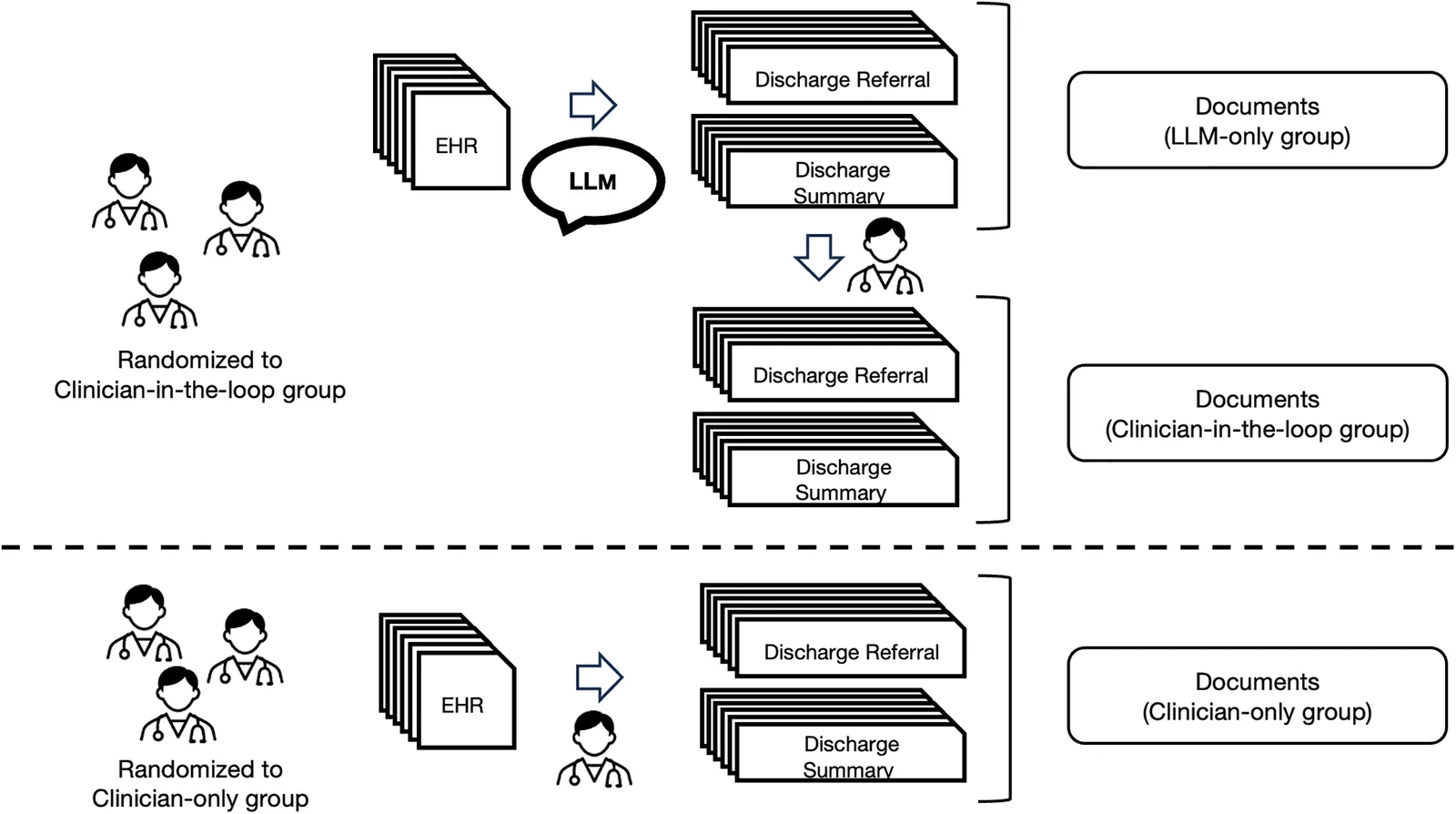
Study arms and clinical-documentation workflow across six simulated cases. Participants were randomized to the Clinician-in-the-loop group or Clinician-only group. Clinician-in-the-loop group: participants used the LLM assistant to generate an initial draft, then edited the draft and submitted the final documents (a discharge summary and a discharge referral); in parallel, the unedited LLM drafts produced before any clinician review were saved and analyzed as the LLM-only group. Clinician-only group: for each of six simulated EHR cases, participants created and submitted the clinical documents directly from the EHR without the LLM assistant.

### Outcome measures

The primary outcome was the average time required to complete each clinical document. Document creation time was automatically recorded within the study web application.

For the secondary outcome, document quality was independently evaluated by five ophthalmologists: Keina S. (Case 1), K.I. (Case 2), M.M. (Case 3), N.U.-A. (Case 4), and Kenji S. (Cases 5 and 6). Cases were matched to each evaluator’s clinical expertise. Evaluators were blinded to participant identity and study-group assignment. Document quality was assessed across six domains:

1. Medical Accuracy: The comprehensiveness of the information provided in the medical record
2. Language: Text style and vocabulary fit the situation and the intended target group that will read the text
3. Conciseness: The text contains only the strictly necessary information, and follows a logical or chronological order
4. Presence of Hallucinations: Any text that is factually incorrect or inconsistent, or unrelated to the medical record
5. Validity for Clinical Use: The text is deemed as usable in a true clinical scenario dealing with real patient information
6. Possibility of Harm: The severity and likelihood of potential harm based on people acting on the text

Domains 1–5 followed the framework of Sánchez-Rosenberg et al. (2024) [16]. Although Sánchez-Rosenberg et al. defined Domain 6 as “possibility of bias,” we instead applied the rubric of Singhal et al. (2023) [29] and adapted Domain 6 to “possibility of harm,” as the former may suggest medical demographic bias, whereas we focused on harmful medical misinformation. Detailed evaluation criteria—following Sánchez-Rosenberg et al. (2024) for Domains 1–5 and Singhal et al. (2023) for Domain 6—are provided in S3 Appendix.

In addition to these domain-based evaluations, each document was subjectively rated on an overall quality scale from 0 to 10. Furthermore, evaluators were asked to guess the study group to which each document belonged.

### Analysis

Although all participants were randomized, four participants withdrew before initiating any study procedures owing to lack of time and provided no evaluable data; they had no exposure to the intervention and were unaware of their assignment. Consequently, analyses were conducted in the evaluable population, defined as all randomized participants with evaluable outcome data, and an intention-to-treat (ITT) analysis was not feasible because of missing data.

For the primary outcome, the mean and 95% confidence intervals were calculated separately for discharge summaries and discharge referrals. Separate linear mixed models (LMMs) were constructed for each document type (i.e., discharge summaries and discharge referrals) to analyze the primary outcome of document creation time. Specifically, group (the Clinician-only group vs. the Clinician-in-the-loop group) and clinical experience level (junior residents vs. all other participants) were included as fixed effects, and a participant-specific random intercept was included to account for repeated document-level observations within participants. As supplementary sensitivity analyses, separate document-level linear regression models with the same fixed effects but without participant-specific random intercepts were fitted to assess the influence of accounting for within-participant correlation on the estimates. As an exploratory analysis in this pilot study, an additional model including the group × clinical experience level interaction was fitted, and its estimates were reported alongside those of the primary models.

For the secondary outcome, descriptive statistics, including the mean for each evaluation domain, were separately calculated for each simulated patient case. Additionally, for the overall quality scale, the mean and 95% confidence intervals were calculated for each study group, and the primary analyses and sensitivity analyses were conducted using the same modeling strategies as for the primary outcome. For the task in which evaluators were asked to guess the group assignment of each document, the corresponding correct identification rates were computed. All analyses were performed using R version 4.3.3. Given the pilot and exploratory nature of this study, no adjustment for multiple testing was performed.

## Results

A total of 21 physicians were randomized (11 to the Clinician-in-the-loop group, 10 to the Clinician-only group). In the Clinician-in-the-loop group, eight participants completed documentation tasks and three withdrew owing to lack of time; in the Clinician-only group, nine completed and one withdrew for the same reason. As group assignment was revealed only after logging in to the study system, dropouts occurred before participants knew their assignment. In total, 17 physicians completed the study and were included in the analysis. The Clinician-in-the-loop group submitted 48 discharge summaries and 48 referrals, and the Clinician-only group submitted 54 of each; 48 unedited LLM drafts were included in the LLM-only group.

Among the 17 participants, 35.3% were junior residents, 41.2% senior residents, and 23.5% attending ophthalmologists, with a mean clinical experience of 5.8 years. Other demographic information is presented in Table 1.

**Table 1:** Baseline participant demographics.

Participant Characteristic	Total (n=17)	Clinician-in-the-loop (n=8)	Clinician-only (n=9)
Sex (Male), No. (%)	12 (70.6)	6 (75.0)	6 (66.7)
Age, mean (SD), y	30.5 (7.8)	30.0 (5.1)	31.0 (10.0)
Position, No. (%)			
Junior Resident	6 (35.3)	3 (37.5)	3 (33.3)
Senior Resident	7 (41.2)	3 (37.5)	4 (44.4)
Attending Ophthalmologist	4 (23.5)	2 (25.0)	2 (22.2)
Physician Experience, mean (SD), y	5.8 (7.8)	5.0 (5.5)	6.4 (9.7)
EHR Use, mean (SD), hours/day	3.7 (2.1)	4.1 (2.3)	3.3 (1.9)
EHR Use, mean (SD), days/week	5.0 (1.8)	4.9 (1.6)	5.1 (2.1)
Generative AI Use, No. (%)			
Never	1 (5.9)	0 (0.0)	1 (11.1)
0–1 days per week	7 (41.2)	2 (25.0)	5 (55.6)
1–3 days per week	1 (5.9)	1 (12.5)	0 (0.0)
3–5 days per week	5 (29.4)	3 (37.5)	2 (22.2)
5–7 days per week	3 (17.6)	2 (25.0)	1 (11.1)

### Primary outcome

The average mean time required to complete discharge summaries and discharge referrals (measured in seconds) is illustrated in Fig 4. For discharge summaries, the Clinician-in-the-loop group demonstrated shorter mean times than the Clinician-only group in five of the six simulated cases. However, for discharge referrals, the Clinician-in-the-loop group required more time than the Clinician-only group in five of the six cases. A shorter mean time was observed only in Case 1. Detailed results are summarized in S1 Table.

**Fig 4:**
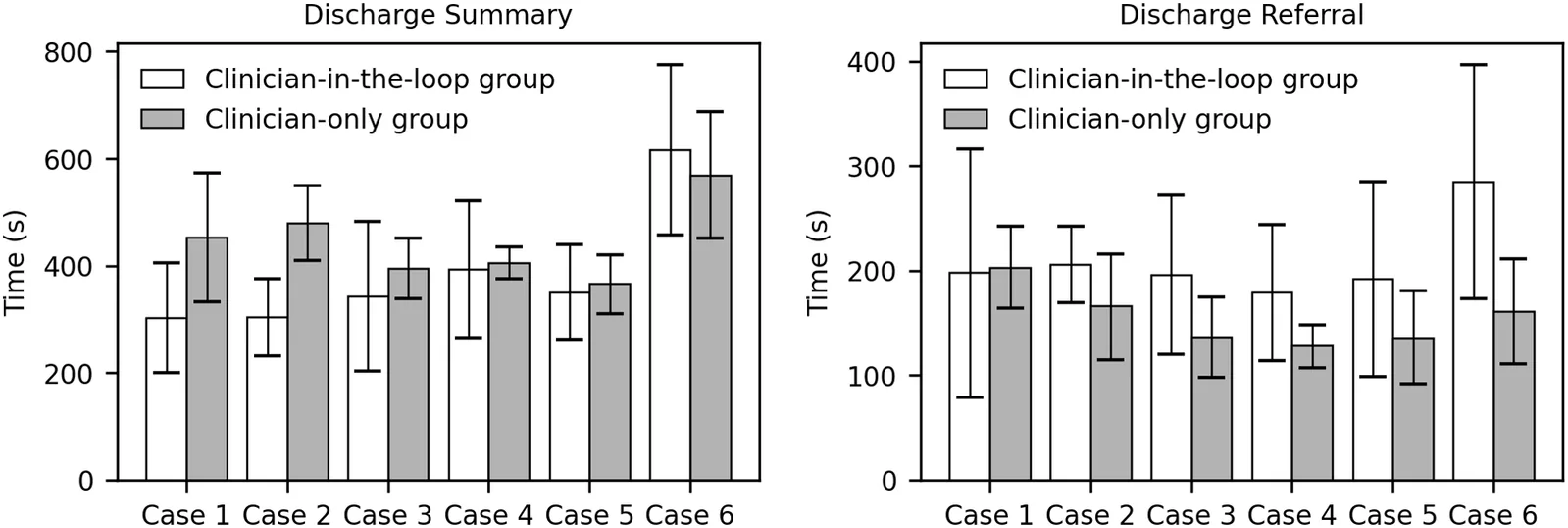
Time to complete clinical documents across six simulated cases. Bar charts show the mean time (seconds) required to complete each document for six simulated patient cases, comparing the Clinician-in-the-loop group (white) with the Clinician-only group (gray). Left: Discharge summaries. Right: Discharge referrals. Error bars indicate 95% confidence intervals.

Separate LMMs and linear regression models were fitted for discharge summaries and referrals to evaluate the effects of group assignment and clinical experience level on document creation time. Full summaries of estimates are presented in Table 2.

**Table 2:** Estimates from linear mixed-effects and linear regression models for document creation time.

Estimates (95% CI) P Value		Without Interaction Terms		With Interaction Terms	
		Linear Mixed-Effects Model	Linear Regression Model	Linear Mixed-Effects Model	Linear Regression Model
**Discharge Summary**	**Intercept**	446.2 (357.2 to 535.2) P < .001	446.2 (388.9 to 503.5) P < .001	441.0 (328.1 to 554.0) P < .001	441.0 (371.0 to 511.0) P < .001
	**Group** ^**a**^	-59.4 (-150.6 to 31.7) P = .18	-59.4 (-118.1 to -0.7) P = .047	-49.1 (-208.8 to 110.6) P = .52	-49.1 (-148.1 to 50.0) P = .33
	**Clinical Experience Level** ^**b**^	-2.3 (-97.4 to 92.9) P = .96	-2.3 (-63.6 to 59.0) P = .94	5.5 (-132.8 to 143.8) P = .93	5.5 (-80.2 to 91.2) P = .90
	**Group × Clinical Experience Level (Interaction)**	–	–	-16.0 (-214.9 to 182.8) P = .86	-16.0 (-139.3 to 107.2) P = .80
**Discharge Referral**	**Intercept**	170.0 (111.5 to 228.6) P < .001	170.0 (137.9 to 202.2) P < .001	149.1 (78.1 to 220.1) P < .001	149.1 (110.6 to 187.6) P < .001
	**Group**	52.9 (-7.0 to 112.9) P = .08	52.9 (20.1 to 85.8) P = .002	94.8 (-5.6 to 195.2) P = .06	94.8 (40.3 to 149.3) P < .001
	**Clinical Experience Level**	-22.3 (-84.8 to 40.3) P = .46	-22.3 (-56.6 to 12.1) P = .20	9.2 (-77.8 to 96.1) P = .82	9.2 (-38.0 to 56.3) P = .70
	**Group × Clinical Experience Level (Interaction)**	–	–	-64.9 (-189.9 to 60.1) P = .28	-64.9 (-132.8 to 2.9) P = .060

a. Group: Clinician-only vs. Clinician-in-the-loop (Clinician-only group was used as the reference)

b. Clinical Experience Level: junior residents vs. all other participants

In the primary analysis for discharge summaries, the Clinician-in-the-loop group assignment was not significantly associated with document creation time compared to the Clinician-only group assignment (β = −59.4, 95% CI: −150.6 to 31.7, p = .18). In the supplementary document-level sensitivity analysis, the Clinician-in-the-loop group was associated with a shorter document creation time (β = −59.4, 95% CI: −118.1 to −0.7, p = .047).

In the primary analysis for discharge referrals, the Clinician-in-the-loop group assignment was not significantly associated with document creation time (β = 52.9, 95% CI: −7.0 to 112.9, p = .08). In the document-level sensitivity analysis, the Clinician-in-the-loop group was associated with a longer document creation time (β = 52.9, 95% CI: 20.1 to 85.8, p = .002).

In the exploratory LMM analysis, the interaction term between group and clinical experience level was not statistically significant (p = .28), although the estimated value was −64.9 seconds (95% CI: −189.9 to 60.1). Similarly, in the exploratory linear regression analysis, although the interaction term was not statistically significant (p = .060), the estimated value was −64.9 seconds (95% CI: −132.8 to 2.9).

### Secondary outcome

The submitted clinical documents were evaluated across six predefined quality domains. The Clinician-in-the-loop group showed higher average scores than the Clinician-only group across nearly all domains in discharge summaries and discharge referrals. The LLM-only group consistently received the lowest scores across most domains for both discharge summaries and discharge referrals; however, in the discharge summaries, the Possibility of Harm and Validity for Clinical Use domains, and in the discharge referrals, the Possibility of Harm domain, achieved scores comparable to those of the Clinician-only group (Fig 5).

**Fig 5:**
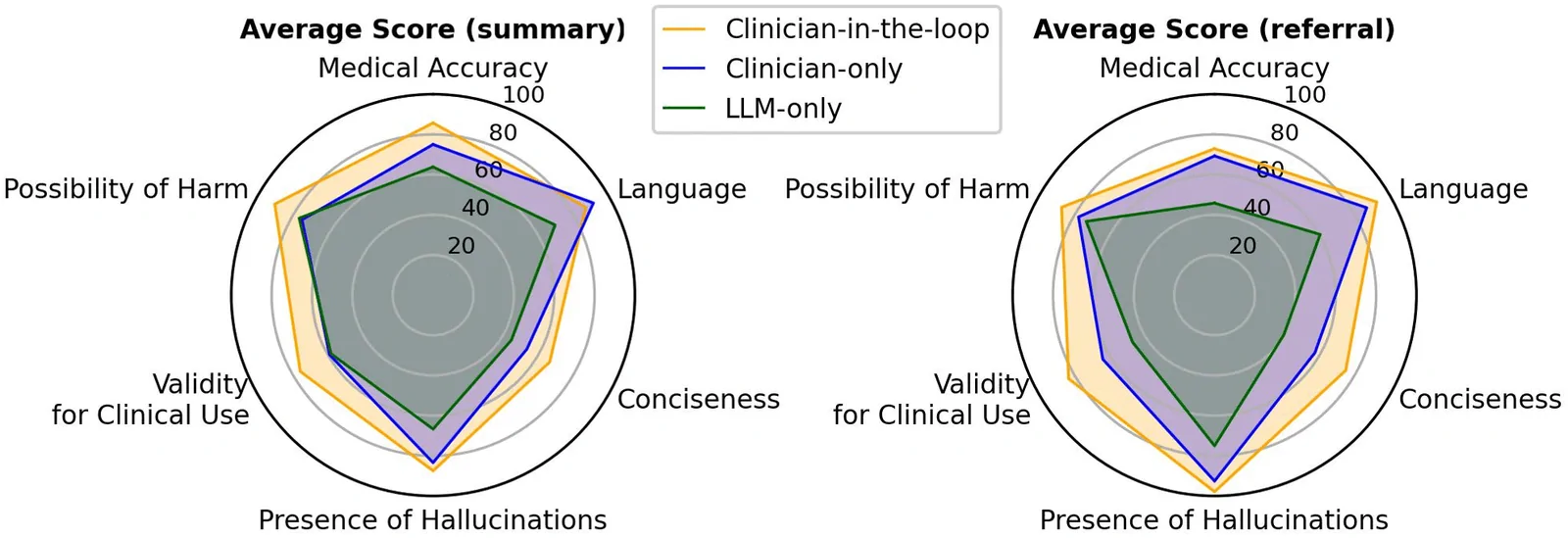
Average evaluation scores across document types by domain. Radar charts displaying the average evaluation scores for each quality domain across three groups (Clinician-in-the-loop, Clinician-only, and LLM-only) for discharge summaries (left) and discharge referrals (right). Scores represent the mean values across all simulated cases, aggregated across six domains: Medical Accuracy, Language, Conciseness, Presence of Hallucinations, Validity for Clinical Use, and Possibility of Harm. Higher scores indicate better performance.

Each document was also rated on an overall quality scale from 0 to 10. The Clinician-in-the-loop group received the highest mean scores for both document types: 7.8 (95% CI: 7.4 to 8.2) for discharge summaries and 8.0 (95% CI: 7.6 to 8.4) for discharge referrals, respectively. Detailed results are summarized in Table 3. Furthermore, separate LMMs and linear regression models were constructed for each document type. In the primary analysis, the Clinician-in-the-loop group had a significantly higher overall quality score than the Clinician-only group for discharge referrals. In the sensitivity analyses, the Clinician-in-the-loop group had significantly higher overall quality scores for both discharge summaries and discharge referrals (see Table 4 for the full summaries of estimates).

**Table 3:** Overall quality ratings of generated documents by group and document type.

Document Type	Group	Mean Score	95% CI
	Clinician-in-the-loop	7.8	7.4 to 8.2
Discharge Summary	Clinician-only	6.9	6.3 to 7.6
	LLM-only	6.0	5.5 to 6.5
	Clinician-in-the-loop	8.0	7.6 to 8.4
Discharge Referral	Clinician-only	6.8	6.2 to 7.4
	LLM-only	5.3	4.9 to 5.8

**Table 4:** Estimates from linear mixed-effects and linear regression models for overall document quality ratings.

Estimates(95% CI)P Value			Without Interaction Terms		With Interaction Terms	
			Linear Mixed-Effects Model	Linear Regression Model	Linear Mixed-Effects Model	Linear Regression Model
Discharge Summary	Intercept		6.85 (6.07 to 7.64) P < .001	6.84 (6.32 to 7.36) P < .001	6.93 (6.06 to 7.80) P < .001	6.93 (6.37 to 7.49) P < .001
	Group^a^	Clinician-in-the-loop	0.84 (-0.24 to 1.91) P = .12	0.83 (0.11 to 1.56) P = .023	0.88 (-0.40 to 2.16) P = .17	0.88 (0.06 to 1.70) P = .037
		Clinician-only (Ref)	–	–	–	–
		LLM-only	-0.96 (-2.03 to 0.12) P = .078	-0.96 (-1.68 to -0.24) P = .010	-1.29 (-2.57 to -0.01) P = .048	-1.29 (-2.11 to -0.47) P = .002
	Clinical Experience Level^b^		0.33 (-0.88 to 1.55) P = .57	0.40 (-0.30 to 1.09) P = .26	-0.01 (-1.85 to 1.83) P = .99	-0.01 (-1.20 to 1.17) P = .98
	Interaction	Clinician-in-the-loop	–	–	-0.13 (-2.76 to 2.50) P = .92	-0.13 (-1.82 to 1.56) P = .88
		Clinician-only (Ref)	–	–	–	–
		LLM-only	–	–	1.37 (-1.26 to 4.00) P = .29	1.37 (-0.32 to 3.06) P = .11
Discharge Referral	Intercept		6.85 (6.30 to 7.40) P < .001	6.84 (6.34 to 7.35) P < .001	6.93 (6.33 to 7.53) P < .001	6.93 (6.38 to 7.48) P < .001
	Group	Clinician-in-the-loop	1.17 (0.41 to 1.93) P = .004	1.17 (0.46 to 1.87) P = .001	1.15 (0.27 to 2.04) P = .013	1.15 (0.35 to 1.96) P = .005
		Clinician-only (Ref)	–	–	–	–
		LLM-only	-1.48 (-2.24 to -0.72) P < .001	-1.48 (-2.18 to -0.77) P < .001	-1.73 (-2.62 to -0.85) P < .001	-1.73 (-2.54 to -0.93) P < .001
	Clinical Experience Level		-0.14 (-0.95 to 0.67) P = .71	-0.13 (-0.81 to 0.55) P = .71	-0.51 (-1.79 to 0.77) P = .42	-0.51 (-1.67 to 0.65) P = .39
	Interaction	Clinician-in-the-loop	–	–	0.10 (-1.73 to 1.92) P = .92	0.10 (-1.56 to 1.75) P = .91
		Clinician-only (Ref)	–	–	–	–
		LLM-only	–	–	1.07 (-0.76 to 2.89) P = .24	1.07 (-0.59 to 2.73) P = .21

a) Group: Clinician-only group was used as the reference

b) Clinical Experience Level: junior residents vs. all other participants

c) Interaction: Group (Clinician-only group was used as the reference) × Clinical Experience Level

For discharge summaries, the Clinician-in-the-loop group was correctly identified in 60.4% of cases, whereas the Clinician-only group was correctly identified in 75.9% of cases. For discharge referrals, the rates were 56.3% for the Clinician-in-the-loop group and 63.0% for the Clinician-only group (S2 Table).

## Discussion

This randomized controlled trial evaluated the utility of LLMs for clinical documentation within a clinician-in-the-loop workflow, explicitly assessing both time and quality outcomes. We compared a clinician-in-the-loop workflow with manual drafting because LLM-only output remains difficult to implement safely in routine clinical practice. Although no statistically significant differences in document creation time were observed in the primary analyses using LMMs, the sensitivity analyses using linear regression models showed a significantly shorter creation time for discharge summaries and a significantly longer creation time for discharge referrals in the Clinician-in-the-loop group. Clinician-edited LLM drafts generally achieved higher scores than manually drafted documents across most quality domains and overall quality ratings. Although the interaction terms were not statistically significant, the exploratory analyses suggested a potential interaction between clinician seniority and the effect of LLM use.

Several studies have compared AI-generated documents with those written by clinicians (Table 5). For relatively simple, short-form clinical texts—such as radiology reports and progress notes—LLM outputs have achieved performance comparable to human-written documents [20]. However, as both the input prompts and target outputs lengthen (e.g., discharge referrals and discharge summaries), known issues such as hallucinations and other errors become more prominent [11,12,15,16]. Consistent with these reports, our LLM-only group produced drafts far more quickly, albeit of inferior quality. In routine clinical care, such deficiencies are unacceptable; accordingly, any LLM-enabled documentation workflow should make clinician review and revision an essential step rather than an optional safeguard. This physician-led use of LLMs is also preferred from the patient’s perspective, as shown by recent large-scale international survey data [30].

**Table 5:** Overview of published studies evaluating LLM interventions for clinical document generation.

Author (Year)	Study Design	Intervention	Target Medical Document			Technique of Prompting
			Discharge Summary	Discharge Referral	Others	
**Current Study**	**RCT**	**LLM-assisted(for time and quality)**	○	○		**Template +** **data extraction**
Mehri et al. (2026) [18]	Comparative Evaluation	LLM-only	○			Prompt Refinement
Schoonbeek et al. (2025) [26]	Comparative Evaluation	LLM-only			○	Prompt Refinement
Williams et al. (2025a) [17]	Comparative Evaluation	LLM-only	○			Prompt Refinement
Williams et al. (2025b) [23]	single arm	LLM-only			○	Prompt Refinement
Ganzinger et al. (2025) [11]	single arm	LLM-only	○			Prompt Refinement
Karino et al. (2025) [12]	Comparative Evaluation	LLM-only	○			No Information
Koh et al. (2025) [10]	single arm	LLM-only	○			No Information
Tung et al. (2024) [19]	Comparative Evaluation	LLM-only		○		Prompt Refinement
Chua et al. (2024) [15]	Comparative Evaluation	LLM-assisted (for time)LLM-only (for quality)	○			Prompt Refinement
Schwieger et al. (2024) [13]	Comparative Evaluation	LLM-only	○			Prompt Refinement
Van Veen et al. (2024) [20]	Comparative Evaluation	LLM-only			○	Fine‑tune / in‑context learning
Clough et al. (2024) [14]	Comparative Evaluation	LLM-only	○			Prompt Refinement
Barak-Corren et al. (2024) [25]	Comparative Evaluation	LLM-assisted (for time)LLM-only (for quality)			○	Prompt Refinement
Sanchez-Rosenberg et al. (2024) [16]	Comparative Evaluation	LLM-only	○	○		Prompt Refinement
Sun et al. (2023) [24]	Comparative Evaluation	LLM-only			○	No Information
Chung et al. (2023) [21]	single arm	LLM-only			○	Prompt Refinement

Two studies have evaluated documentation time in clinician-in-the-loop workflows—one focused on discharge summaries and one on supervisory notes [15,25]. Both reported reduced completion time. However, neither study assessed document quality; therefore, whether efficiency gains were achieved at the expense of accuracy or completeness remains unresolved. This study jointly evaluates documentation time and quality within a clinician-in-the-loop workflow, using a randomized controlled design, and assesses its practical applicability within clinical documentation practice. Across multiple quality domains, the clinician-in-the-loop approach outperformed manual drafting, indicating that this method is not merely “quick and dirty” but instead improves quality while potentially alleviating documentation burden for some document types, as suggested by the sensitivity analysis. One potential mechanism underlying these findings is that, by generating an initial draft, the LLM supports structured information retrieval and organization from the electronic health record. Clinicians then review and revise this draft rather than composing the document from scratch, which enables more critical appraisal of the content while reducing the cognitive burden associated with information retrieval, structuring, and writing. Taken together, these findings suggest that the clinician-in-the-loop approach may support documentation workflows and deserves further evaluation in broader clinical settings.

In this study, we implemented an extraction-based system and deployed it as an LLM assistant, reflecting the evidence that LLM data extraction can be highly accurate [31–33]. The differing directions of the estimated effects on completion time between discharge summaries and discharge referrals may reflect differences in document structure. For highly standardized documents such as discharge summaries, direct extraction into fixed templates may be sufficient. By contrast, discharge referrals involve greater narrative variability, and documents created from rigid templates often require additional modification to fit individual physician preferences. When creating documents with such narrative diversity, providing support for flexible, clinician-authored template creation alongside structured data extraction may be more appropriate and warrants further evaluation in real-world clinical settings.

We also examined heterogeneity by clinician seniority during LLM use. The interaction between clinician experience and the effects of AI assistance has been investigated in the recognition of radiological and pathological images [34–39], whereas, to our knowledge, no such evidence has been available in the context of LLMs. The exploratory analysis suggested possible effect heterogeneity related to clinical experience level, although the interaction terms were not statistically significant. This finding raises the hypothesis that clinical expertise may influence the extent to which clinicians benefit from LLM-assisted documentation workflows. Further studies with larger sample sizes are needed to confirm whether clinician experience modifies the effect of clinician-in-the-loop LLM workflows. Beyond documentation, AI in medicine now extends to areas such as clinical reasoning [40], image recognition [41,42], and basic research [43], and it is important to investigate whether similar patterns are observed in these domains.

This study has several limitations. First, this study was conducted in a single center and a single specialty using a small number of simulated ophthalmology cases, a limited number of participants, and Japanese-language records. While this design allowed us to standardize inputs and evaluate feasibility in a controlled setting, it may not fully capture the complexity, incompleteness, and variability of daily clinical documentation. Documentation structure and workflow also vary across medical specialties. For example, internal medicine often involves comprehensive laboratory and multimodal diagnostic data, intensive care documentation is commonly organized by organ system, and emergency medicine may rely on fragmented, time-sequenced records. Although the assistant is designed for structured extraction and template-based drafting and could in principle be adapted using specialty-specific templates, empirical validation across other clinical contexts is required. The assistant’s performance may also differ across languages and model versions. Larger confirmatory studies using real patient data across multiple institutions, specialties, and languages are needed. Second, as templates were authored by an investigator rather than participants, they may not fully reflect individual writing styles. Although we derived templates from recent discharge summaries and referrals in our ophthalmology department to minimize stylistic mismatch, inter-physician variability may have influenced results. Future studies should evaluate the LLM system using clinician-authored, personalized templates prior to testing. Third, because of the pilot nature of the study and the small sample size, the present findings—including those on documentation time and on effect modification by clinical experience level—should be interpreted as hypothesis-generating rather than confirmatory. In particular, both the effects on documentation time and the estimated effect modification by clinical experience level may vary according to document type and model specification and therefore require confirmation in future larger studies. Fourth, to standardize task conditions across participants, both the task order and case sequence were fixed in this study. However, this design may have introduced learning effects, fatigue effects, or carryover effects between tasks. In addition, because all participants processed the same cases in the same sequence, the effects of case-specific difficulty could not be formally separated from order-related effects; therefore, formal exploratory analyses of order-related trends were not performed in this pilot design. Future larger studies should consider randomization or counterbalancing of task order and case sequence. Fifth, document quality for each case was evaluated by a single expert ophthalmologist who was blinded to participant identity and study group. Although a previously published evaluation framework and standardized scoring criteria were used to promote consistency, duplicate evaluations and adjudication by multiple reviewers were not performed, and inter-rater agreement and reliability could not therefore be formally assessed; the reproducibility of the quality ratings thus remains uncertain. Future studies should incorporate multiple independent reviewers, duplicate assessments, and inter-rater reliability analyses.

In conclusion, a clinician-in-the-loop workflow was generally associated with improved overall document quality and showed potential for shorter documentation time for certain document types, although no statistically significant difference in documentation time was observed in the primary analysis. These findings highlight the importance of active clinician supervision when integrating LLMs into clinical documentation workflows. Further studies in broader clinical settings are warranted to evaluate the generalizability and practical applicability of this approach.

## Supporting information

S1 CONSORT Checklist CONSORT 2025 Checklist. Hopewell S, Chan AW, Collins GS, Hróbjartsson A, Moher D, Schulz KF, et al. CONSORT 2025 explanation and elaboration: updated guideline for reporting randomised trials. BMJ. 2025 Apr 14;e081124. https://doi.org/10.1136/bmj-2024-081124. This checklist is licensed under the Creative Commons Attribution 4.0 International License (CC BY 4.0; https://creativecommons.org/licenses/by/4.0/).
(DOCX)

S1 Protocol Protocol. (PDF)

S1 Appendix The six simulated patient records. (PDF)

S2 Appendix The templates for the discharge summary and the discharge referral. (PDF)

S3 Appendix Evaluation criteria. (PDF)

S1 Table Mean Document Creation Time by Case and Group. (DOCX)

S2 Table Distribution of Predicted Group Assignments by Actual Study Group (%). (DOCX)

## References

[1] Sinsky C, Colligan L, Li L, Prgomet M, Reynolds S, Goeders L. Allocation of physician time in ambulatory practice: a time and motion study in 4 specialties. Ann Intern Med. 2016;165(11):753–60. doi: 10.7326/M16-0961 27595430

[2] Yamaguchi N, Mochida Y, Matsubara S, Sumitani S. Factors contributing to physician overwork in 80 Saiseikai hospitals of Japan. 2020. doi: 10.21203/rs.3.rs-15702/v1

[3] West CP, Dyrbye LN, Shanafelt TD. Physician burnout: contributors, consequences and solutions. J Intern Med. 2018;283(6):516–29. doi: 10.1111/joim.12752 29505159

[4] Rao SK, Kimball AB, Lehrhoff SR, Hidrue MK, Colton DG, Ferris TG, et al. The Impact of Administrative Burden on Academic Physicians: Results of a Hospital-Wide Physician Survey. Acad Med. 2017;92(2):237–43. doi: 10.1097/ACM.0000000000001461 28121687

[5] Gardner RL, Cooper E, Haskell J, Harris DA, Poplau S, Kroth PJ. Physician stress and burnout: the impact of health information technology. J Am Med Inform Assoc. 2019;26(2):106–14. doi: 10.1093/jamia/ocy145 PMC7647171 30517663

[6] Gesner E, Dykes PC, Zhang L, Gazarian P. Documentation Burden in Nursing and Its Role in Clinician Burnout Syndrome. Appl Clin Inform. 2022;13(5):983–90. doi: 10.1055/s-0042-1757157 36261113 PMC9581587

[7] Omiye JA, Gui H, Rezaei SJ, Zou J, Daneshjou R. Large Language Models in Medicine: The Potentials and Pitfalls: A Narrative Review. Ann Intern Med. 2024;177(2):210–20. doi: 10.7326/M23-2772 38285984

[8] Zheng Y, Gan W, Chen Z, Qi Z, Liang Q, Yu PS. Large Language Models for Medicine: A Survey. arXiv. 2024. http://arxiv.org/abs/2405.13055

[9] Bedi S, Liu Y, Orr-Ewing L, Dash D, Koyejo S, Callahan A. A systematic review of testing and evaluation of healthcare applications of large language models (LLMs). 2024. doi: 10.1101/2024.04.15.24305869 PMC11480901 39405325

[10] Koh MCY, Ngiam JN, Oon JEL, Lum LH-W, Smitasin N, Archuleta S. Using ChatGPT for writing hospital inpatient discharge summaries - perspectives from an inpatient infectious diseases service. BMC Health Serv Res. 2025;25(1):221. doi: 10.1186/s12913-025-12373-w 39924512 PMC11809107

[11] Ganzinger M, Kunz N, Fuchs P, Lyu CK, Loos M, Dugas M, et al. Automated generation of discharge summaries: leveraging large language models with clinical data. Scientific Reports. 2025;15(1). doi: 10.1038/s41598-025-01618-7 PMC12069548 40355506

[12] Karino M, So C, Jinta T, Tanaka M, Nakamura T, Okafuji K, et al. Exploring the potential of ChatGPT for the summarization of patient medical histories: A pilot study. Cureus. 2025. doi: 10.7759/cureus.84133 PMC12168705 40525027

[13] Schwieger A, Angst K, de Bardeci M, Burrer A, Cathomas F, Ferrea S, et al. Large language models can support generation of standardized discharge summaries - A retrospective study utilizing ChatGPT-4 and electronic health records. Int J Med Inform. 2024;192:105654. doi: 10.1016/j.ijmedinf.2024.105654 39437512

[14] Clough RAJ, Sparkes WA, Clough OT, Sykes JT, Steventon AT, King K. Transforming healthcare documentation: harnessing the potential of AI to generate discharge summaries. BJGP Open. 2024;8(1):BJGPO.2023.0116. doi: 10.3399/BJGPO.2023.0116 37699649 PMC11169980

[15] Chua CE, Lee Ying Clara N, Furqan MS, Lee Wai Kit J, Makmur A, Tham YC, et al. Integration of customised LLM for discharge summary generation in real-world clinical settings: a pilot study on RUSSELL GPT. Lancet Reg Health West Pac. 2024;51:101211. doi: 10.1016/j.lanwpc.2024.101211 39881943 PMC11776078

[16] Sánchez-Rosenberg G, Magnéli M, Barle N, Kontakis MG, Müller AM, Wittauer M, et al. ChatGPT-4 generates orthopedic discharge documents faster than humans maintaining comparable quality: a pilot study of 6 cases. Acta Orthop. 2024;95:152–6. doi: 10.2340/17453674.2024.40182 38597205 PMC10959013

[17] Williams CYK, Subramanian CR, Ali SS, Apolinario M, Askin E, Barish P, et al. Physician- and large language model–generated hospital discharge summaries. JAMA Internal Medicine. 2025;185(7):818. doi: 10.1001/jamainternmed.2025.0821 PMC12053800 40323616

[18] Mehri T, Nadalini T, Hoekman AH, van der Laan TP, Kagialari K, Wagner RK, et al. Assessing the quality of AI-generated and physician-written discharge summaries: evaluation of an EHR-integrated tool in a Dutch academic hospital. EBioMedicine. 2026;127:106247. doi: 10.1016/j.ebiom.2026.106247 41962200 PMC13091380

[19] Tung JYM, Gill SR, Sng GGR, Lim DYZ, Ke Y, Tan TF, et al. Comparison of the Quality of Discharge Letters Written by Large Language Models and Junior Clinicians: Single-Blinded Study. J Med Internet Res. 2024;26:e57721. doi: 10.2196/57721 39047282 PMC11306941

[20] Van Veen D, Van Uden C, Blankemeier L, Delbrouck J-B, Aali A, Bluethgen C, et al. Adapted large language models can outperform medical experts in clinical text summarization. Nat Med. 2024;30(4):1134–42. doi: 10.1038/s41591-024-02855-5 38413730 PMC11479659

[21] Chung EM, Zhang SC, Nguyen AT, Atkins KM, Sandler HM, Kamrava M. Feasibility and acceptability of ChatGPT generated radiology report summaries for cancer patients. Digit Health. 2023;9. doi: 10.1177/20552076231221620 PMC10734360 38130802

[22] Zaretsky J, Kim JM, Baskharoun S, Zhao Y, Austrian J, Aphinyanaphongs Y, et al. Generative Artificial Intelligence to Transform Inpatient Discharge Summaries to Patient-Friendly Language and Format. JAMA Netw Open. 2024;7(3):e240357. doi: 10.1001/jamanetworkopen.2024.0357 38466307 PMC10928500

[23] Williams CYK, Bains J, Tang T, Patel K, Lucas AN, Chen F. Evaluating large language models for drafting emergency department encounter summaries. PLOS Digital Health. 2025;4(6):e0000899. doi: 10.1371/journal.pdig.0000899 PMC12173386 40526634

[24] Sun Z, Ong H, Kennedy P, Tang L, Chen S, Elias J. Evaluating GPT4 on Impressions Generation in Radiology Reports. Radiology. 2023;307(5). doi: 10.1148/radiol.231259 PMC10534271 37367439

[25] Barak-Corren Y, Wolf R, Rozenblum R, Creedon JK, Lipsett SC, Lyons TW, et al. Harnessing the Power of Generative AI for Clinical Summaries: Perspectives From Emergency Physicians. Ann Emerg Med. 2024;84(2):128–38. doi: 10.1016/j.annemergmed.2024.01.039 38483426

[26] Schoonbeek RC, Workum JD, Schuit SCE, Hoekman AH, Mehri T, Doornberg JN, et al. Quality and efficiency of integrating customised large language model-generated summaries versus physician-written summaries: a validation study. BMJ Open. 2025;15(9):e099301. doi: 10.1136/bmjopen-2025-099301 40908007 PMC12414186

[27] Al-Garadi M, Mungle T, Ahmed A, Sarker A, Miao Z, Matheny ME. Large Language Models in Healthcare. https://arxiv.org/abs/2503.04748. 2025. Accessed 2025 July 25.

[28] Hopewell S, Chan AW, Collins GS, Hróbjartsson A, Moher D, Schulz KF. CONSORT 2025 explanation and elaboration: updated guideline for reporting randomised trials. BMJ. 2025;:e081124. doi: 10.1136/bmj-2024-081124 PMC11995452 40228832

[29] Singhal K, Azizi S, Tu T, Mahdavi SS, Wei J, Chung HW, et al. Large language models encode clinical knowledge. Nature. 2023;620(7972):172–80. doi: 10.1038/s41586-023-06291-2 37438534 PMC10396962

[30] Busch F, Hoffmann L, Xu L, Zhang LJ, Hu B, García-Juárez I, et al. Multinational Attitudes Toward AI in Health Care and Diagnostics Among Hospital Patients. JAMA Netw Open. 2025;8(6):e2514452. doi: 10.1001/jamanetworkopen.2025.14452 40493367 PMC12152705

[31] Huang J, Yang DM, Rong R, Nezafati K, Treager C, Chi Z, et al. A critical assessment of using ChatGPT for extracting structured data from clinical notes. NPJ Digit Med. 2024;7(1):106. doi: 10.1038/s41746-024-01079-8 38693429 PMC11063058

[32] Ntinopoulos V, Rodriguez Cetina Biefer H, Tudorache I, Papadopoulos N, Odavic D, Risteski P, et al. Large language models for data extraction from unstructured and semi-structured electronic health records: a multiple model performance evaluation. BMJ Health Care Inform. 2025;32(1):e101139. doi: 10.1136/bmjhci-2024-101139 39832824 PMC11751965

[33] Keloth VK, Selek S, Chen Q, Gilman C, Fu S, Dang Y. Social determinants of health extraction from clinical notes across institutions using large language models. NPJ Digit Med. 2025;8(1). doi: 10.1038/s41746-025-01645-8 PMC12084648 40379919

[34] Yu F, Moehring A, Banerjee O, Salz T, Agarwal N, Rajpurkar P. Heterogeneity and predictors of the effects of AI assistance on radiologists. Nat Med. 2024;30(3):837–49. doi: 10.1038/s41591-024-02850-w 38504016 PMC10957478

[35] Ahn JS, Ebrahimian S, McDermott S, Lee S, Naccarato L, Di Capua JF, et al. Association of Artificial Intelligence-Aided Chest Radiograph Interpretation With Reader Performance and Efficiency. JAMA Netw Open. 2022;5(8):e2229289. doi: 10.1001/jamanetworkopen.2022.29289 36044215 PMC9434361

[36] Gaube S, Suresh H, Raue M, Lermer E, Koch TK, Hudecek MFC. Scientific Reports. 2023;13(1):1383. doi: 10.1038/s41598-023-28633-w PMC9876883 36697450

[37] Bulten W, Balkenhol M, Belinga J-JA, Brilhante A, Çakır A, Egevad L, et al. Artificial intelligence assistance significantly improves Gleason grading of prostate biopsies by pathologists. Mod Pathol. 2021;34(3):660–71. doi: 10.1038/s41379-020-0640-y 32759979 PMC7897578

[38] Kim HJ, Choi WJ, Gwon HY, Jang SJ, Chae EY, Shin HJ, et al. Improving mammography interpretation for both novice and experienced readers: a comparative study of two commercial artificial intelligence software. Eur Radiol. 2024;34(6):3924–34. doi: 10.1007/s00330-023-10422-8 37938383

[39] Dratsch T, Chen X, Rezazade Mehrizi M, Kloeckner R, Mähringer-Kunz A, Püsken M. Automation Bias in Mammography: The Impact of Artificial Intelligence BI-RADS Suggestions on Reader Performance. Radiology. 2023;307(4):e222176. doi: 10.1148/radiol.222176 37129490

[40] Goh E, Gallo R, Hom J, Strong E, Weng Y, Kerman H, et al. Large Language Model Influence on Diagnostic Reasoning: A Randomized Clinical Trial. JAMA Netw Open. 2024;7(10):e2440969. doi: 10.1001/jamanetworkopen.2024.40969 39466245 PMC11519755

[41] Tomita K, Nishida T, Kitaguchi Y, Kitazawa K, Miyake M. Image Recognition Performance of GPT-4V(ision) and GPT-4o in Ophthalmology: Use of Images in Clinical Questions. Clin Ophthalmol. 2025;19:1557–64. doi: 10.2147/OPTH.S494480 40357454 PMC12068282

[42] Kamei T, Miyake M, Sado K, Morino K, Mori Y, Tabara Y, et al. Association between the retinal age gap and systemic diseases in the Japanese population: the Nagahama study. Jpn J Ophthalmol. 2025;69(4):616–23. doi: 10.1007/s10384-025-01205-3 40304887 PMC12339582

[43] Hosoda Y, Miyake M, Meguro A, Tabara Y, Iwai S, Ueda-Arakawa N, et al. Keratoconus-susceptibility gene identification by corneal thickness genome-wide association study and artificial intelligence IBM Watson. Commun Biol. 2020;3(1):410. doi: 10.1038/s42003-020-01137-3 32737415 PMC7395727

