# Supplementary material for: Evaluating an LLM-assisted workflow for clinical documentation: A pilot randomized controlled trial on time and quality
Source: PLOS Digit Health. 2026 Oct 26;5(10):e0001799. doi: 10.1371/journal.pdig.0001799 (PMC13704812; doi:10.1371/journal.pdig.0001799)
Supplement: Supplementary file 3 [file pdig.0001799.s004.pdf]

The Predefined Templates

Here we describe the Predefined Templates supplied to the LLM assistant, CocktailAI. Although templates would ordinarily be authored by end users, in this study they were prepared in advance by a study team member (T.T.) who was not involved in creating the simulated patient records, in order to reduce participant burden. The templates follow these rules:

- Segments enclosed in {…} are machine-readable directives. The LLM assistant interprets these markers and extracts the specified information directly from the electronic health record (EHR).
- Nesting braces (i.e., {…{…}}) specifies how the extraction should be performed (e.g., required formats, scope, or selection criteria).
- The form {prompt? {…}} passes the inner text to the LLM assistant as a prompt. Use this when you want a flexible, generated response rather than literal data extraction.

Document Type	Templates
Discharge Summary	Admission Date: {Admission Date {Write in the form of "yyyy Year mm Month dd Day".}}<br>Discharge Date: {Discharge Date {Write in the form of "yyyy Year mm Month dd Day".}}<br>Discharge Diagnosis:<br>{Diagnosis {Extract all diagnoses.}}<br>Allergies:<br>{Allergy history}<br><br>Chief Complaint: {Chief complaint for this admission, or reason for hospitalization}<br>History of Present Illness: {Present illness {Write in the form of "yyyy Year mm Month dd Day," and specify Right Eye = R), Left Eye = L), Both Eyes = B).}}<br>Ophthalmological Medical History: {Ophthalmological medical history}<br>Systemic Medical History: {Systemic medical history}<br>Regular Medications: {Eye drops and oral medications at the time of admission.}<br><br>Hospital Course: {Prompt? {Briefly describe the course during hospitalization, including surgeries and procedures, using standard style.}}<br><br>Discharge Medications: {Discharge medications {List all medications continued from admission to discharge, as well as any new medications added during hospitalization.}}<br><br>Condition at Discharge: {Condition of the patient at discharge.}<br><br>Discharge Plan: {Discharge plan {Briefly describe the post-discharge plan.}}
Discharge Referral	We would like to inform you that the patient referred by your institution has been discharged.<br><br>On {Surgery date {Write in the format of "yyyy Year mm Month dd Day."}}, we performed {Surgical procedure} for {Diagnosis}.<br>{Postoperative course until discharge {Rewrite the text in a polite style.}}<br>While we will continue to follow up with the patient at our department, we kindly ask for your continued follow-up during their visits to your institution.<br>We sincerely appreciate your referral of this valuable case.<br>We look forward to working with you in the future.
Discharge Referral (No surgery)	We would like to inform you that the patient referred by your institution has been discharged.

	The patient was admitted on {Admission date {Write in the format of "yyyy Year mm Month dd Day."}} and received {Details of treatment during hospitalization} for {Diagnosis} at our hospital.<br>{Course from admission to discharge {Rewrite the text in a polite style, using "desu/masu" tone.}}<br>While we will continue to follow up with the patient at our department, we kindly ask for your continued follow-up during their visits to your institution.<br>We sincerely appreciate your referral of this valuable case.<br>We look forward to working with you in the future.
