# Supplementary material for: Evaluating an LLM-assisted workflow for clinical documentation: A pilot randomized controlled trial on time and quality
Source: PLOS Digit Health. 2026 Oct 26;5(10):e0001799. doi: 10.1371/journal.pdig.0001799 (PMC13704812; doi:10.1371/journal.pdig.0001799)
Supplement: Supplementary file 6 [file pdig.0001799.s006.docx]

| **S1 Table. Mean Document Creation Time by Case and Group** | | | | |
| --- | --- | --- | --- | --- |
| Document Type | Case | Mean Seconds (95%CI) | | |
|  |  | Clinician-in-the-loop group | Clinician-only group | LLM-only group |
|  | Case 1 | **303 (201 to 406)** | 453 (332 to 573) | 7.42 (4.39 to 10.5) |
|  | Case 2 | **304 (232 to 376)** | 480 (410 to 550) | 5.76 (3.80 to 7.73) |
| Discharge Summary | Case 3 | **343 (204 to 483)** | 395 (338 to 452) | 6.05 (4.24 to 7.87) |
|  | Case 4 | **394 (266 to 521)** | 406 (376 to 435) | 7.16 (5.18 to 9.14) |
|  | Case 5 | **351 (263 to 440)** | 366 (311 to 420) | 5.20 (3.73 to 6.68) |
|  | Case 6 | 617 (457 to 776) | **569 (451 to 687)** | 6.48 (4.45 to 8.50) |
|  | Case 1 | **198 (79.3 to 316)** | 203 (164 to 242) | 2.86 (1.74 to 3.97) |
|  | Case 2 | 206 (169 to 242) | **166 (115 to 216)** | 3.94 (2.44 to 5.43) |
| Discharge Referral | Case 3 | 196 (120 to 272) | **137 (98.3 to 175)** | 4.05 (2.07 to 6.03) |
|  | Case 4 | 179 (114 to 244) | **128 (107 to 148)** | 4.36 (3.15 to 5.56) |
|  | Case 5 | 192 (98.8 to 285) | **136 (92.0 to 181)** | 2.83 (2.04 to 3.63) |
|  | Case 6 | 285 (173 to 397) | **161 (111 to 211)** | 3.80 (3.25 to 4.35) |
