# Supplementary material for: Evaluating an LLM-assisted workflow for clinical documentation: A pilot randomized controlled trial on time and quality
Source: PLOS Digit Health. 2026 Oct 26;5(10):e0001799. doi: 10.1371/journal.pdig.0001799 (PMC13704812; doi:10.1371/journal.pdig.0001799)
Supplement: Supplementary file 7 [file pdig.0001799.s007.docx]

| **S2 Table. Distribution of Predicted Group Assignments by Actual Study Group (%)** | | | | |
| --- | --- | --- | --- | --- |
| **Document Type** | **Actual Study Group** | **Predicted Group Assignment (%)** | | |
|  |  | **Clinician-in-the-loop** | **Clinician-only** | **LLM-only** |
|  | Clinician-in-the-loop | 60.4% (29/48) | 20.8% (10/48) | 18.8% (9/48) |
| Discharge Summary | Clinician-only | 16.7% (9/54) | 75.9% (41/54) | 7.4% (4/54) |
|  | LLM-only | 22.9% (11/48) | 2.1% (1/48) | 75.0% (36/48) |
|  | Clinician-in-the-loop | 56.3% (27/48) | 29.2% (14/48) | 14.6% (7/48) |
| Discharge Referral | Clinician-only | 18.5% (10/54) | 63.0% (34/54) | 18.5% (10/54) |
|  | LLM-only | 20.8% (10/48) | 0.0% (0/48) | 79.2% (38/48) |
